# Role of Biatrial Pacing in Prevention of Atrial Fibrillation after Coronary Artery Bypass Surgery

**Published:** 2005-01-01

**Authors:** Massoud Eslami, Hamid S. Mirkhani, Mehdi Sanatkar, Homeira Bayat, Roya Sattarzadeh, Mahmood Mirhoseini

**Affiliations:** * Cardilogy Department, Imam Khomeini Medical Center, Tehran University Of Medical Sciences; † Cardiovascular Surgery Department, Imam Khomeini Medical Center, Tehran University Of Medical Sciences

**Keywords:** fibrillation, pacing, arrhythmia, coronary bypass

## Abstract

**Background:**

Atrial fibrillation (AF) after coronary artery bypass graft surgery (CABG) constitutes the most common sustained arrhythmia and results in prolonged hospitalization. The purpose of this study was to assess simultaneous right and left atrial pacing as prophylaxis for postoperative atrial fibrillation.

**Methods and Results:**

From July 2003 to May 2004, 120 patients without structural heart disease and who underwent CABG were randomly classified into one of the following 3 groups: biatrial pacing (BAP), left atrial pacing (LAP), and no pacing (control). Atrial pacing was performed for 4 days. Post-CABG AF was significantly reduced in BAP group compared to single-site and control group (BAP, 17.5%; LAP, 30%; control, 45%; p=0.02). The mean length of hospital stay was significantly reduced in BAP group. Hospital charges were not significantly different between three groups. The mean length of hospital stay was most significantly reduced in BAP group (6.1±1.2 versus 9.0±4.1 days in the control groups; p=0.002, and 8.7±1.3 days in LAP groups; p=0.01). The mean length of stay in the intensive care unit was also significantly reduced in the BAP group (2.8±0.7 versus 4.6±4.5 days in control group; p=0.04, and 4.2±3.2 days in LAP group; p=0.01).

**Conclusion:**

Simultaneous right and left atrial pacing is well tolerated and is more effective in preventing post-CABG AF than single-site pacing, and, results in a shortened hospital stay. Identifying patients at risk for developing postoperative AF and using this prophylactic method may be the optimal effective strategy.

## Introduction

Atrial fibrillation (AF) after coronary artery bypass surgery (CABG) constitutes the most common sustained arrhythmia [1, 2]. Postoperative atrial fibrillation occurs in 10% to 40% of patients undergoing open-heart surgery [3, 4]. Postoperative AF is associated with stroke and a prolonged hospital stay [5]. Although AF rarely cause serious problem, it can cause considerable discomfort for the patient [6]. Similary, there is controversy regarding methods of preventing postoperative AF. Beta-adrenergic receptor blockers and Class III antiarhythmic agents such as Amiodarone confer some benefits in preventing post-CABG AF, but its incidence is still as high as 30% with these therapies [7, 8]. Preoperative and postoperative digoxin was recommended as an effective preventive method by one group [9]but it was rejected by others [10]; while in one trial, only the combination of beta-blocker and digoxin was found to be effective [11]. The pathogenesis of postoperative AF remains unclear and is presumably multifactorial. Fuller’s study showed the close association with the patient’s age and male gender [3]. Multivariate analysis showed no relationship with the aortic cross-clamp time, the volume of cardioplegia, the number of grafts, the presence of postoperative infarct, or the postoperative CK_MB level [12], although two studies have found a relationship between postoperative AF and the length of the operation [13]. Previous studies demonstrated that arrhythmia was caused by operative damage to the atrial myocardium, so one would expect AF to occur immedietly after the operation. By contrast, AF develops most frequently on the second postoperative day. These observations suggest a different mechanism, such as inflamatory response with atrial edema, pericarditis, or reperfusion injury, rather than a direct ischemic insult [6, 14]. The transient nature of this problem when seen after cardiac surgery suggests a reversible trigger; abnormal automaticity and atrial conduction delay are possible electrophysiological substrates. These would result in the occurrence of atrial ectopy and prolonged atrial activation, with lengthening of the P wave recorded by the ECG [7]. However, a signal averaged ECG of the P wave, which is a measure of regions of delayed atrial activation, is only moderately sensitive in predicting AF after CABG [15]. Signal-average ECG P-wave dispersion has also been recently advocated as a novel measurement of the heterogeneity of atrial depolarization [16].

Biatrial pacing has been shown to be effective in preventing AF after CABG [17, [18]. The purpose of this prospective study was to evaluate the efficacy of biatrial pacing as a prophylactic measure against AF after CABG when compared with no (control) or single- site atrial pacing.

## Materials and Methods

From July 2003 to May 2004, 120 patients that candidate for CABG were enrolled. The study design was approved by the Imam Khomeini Medical Center Committee and was a double-blind protocol in which the surgical staff and principal investigators and the patient were unaware of the assigned pacing modality. Clinical data, lead parameters, and Holter data were collected and recorded into the database by independent blinded investigators. Patients were randomly assigned in a double-blind fashion immediately after surgery to 1 of 3 pacing modes: biatrial pacing (BAP), left atrial pacing (LAP), and no pacing (control). Inclusion criteria consisted of informed consent, age > 50 years, elective heart surgery requiring cardiopulmonary bypass, and normal sinus rhythm. Exclusion criteria included participation in another investigational protocol, presence of a permanent pacemaker, or use antiarrhythmic therapy other than beta-blocker, history of supraventricular (including atrial flutter or AF) or ventricular tachyarrhythmias, redo-operation, patients with valvular heart surgery, and cardiogenic shock. All medications, including beta-blocker and digitalis, were continued until surgery. Postoperatively, these medications were used according to clinical indications. Coronary artery bypass surgery was performed in all patients. Patients underwent CABG on standard cardiopulmonary bypass with myocardial protection provided by blood cardioplegia

After completion of the surgical procedure, 2 temporary unipolar epicardial leads (model 6500, Medtronic, Inc) were attached to the anterior-superior aspect of the right atrium, and a second pair of epicardial leads was attached to the posterior-inferior aspect of the left atrium between the coronary sinus and the right inferior pulmonary vein [19]. The lead was a multifilament, braided, stainless steel. This electrode was attached to a curved needle with polypropylene suture, and the proximal end of the suture was coiled to reduce dislodgment. Biatrial pacing was achieved by simultaneous bipolar pacing of the right and left atria. In patients assigned to LAP group the left atrial pair of unipolar leads was connected to an external pacemaker generator (Pacesetter, Inc), and the proximal ends of the right atrial epicardial pacing electrodes were taped to the chest wall. Sensitivity of the pacing was set at 0.25 mV. The lowest rate was 90 beats per minute and the maximum pacing rate allowed was 120 beats per minute. Overdrive pacing was continued for 4 days, with continuous telemetry monitoring. The pacing and sensing thresholds were checked daily, and the output was adjusted accordingly. The 12-lead ECG was performed daily for 4 days at baseline and during pacing. The pacing wires were removed by simple transcutaneous retraction by 6 day in the absence of a clinical end point. Patients were re-evaluated in the cardiac surgery outpatient clinic 4 weeks after surgery.

## Statistical Analysis

Continuos variables were expressed as mean ± SD. Continuous variables were compared by means of ANOVA tests and discrete variables were compared using the χ^2^ test. p<0.05 was considered statistically significant.

## Results

The patients in this study comprised 40 in BAP group, 40 in LAP group, and 40 patients in control group, respectively. The mean age in each group were BAP, 61.2±6.2; LAP, 62.6±7.2; and control, 60±7.5; p=0.1; 60% in BAP, 57.5% in LAP and 60% in control group were male. All clinical characteristics in each group were similar and well matched (Table 1).

**Figure 1:**
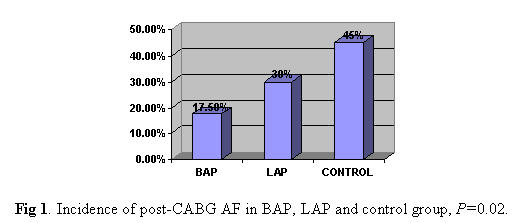
Incidence of post-CABG AF in BAP, LAP and control group, P=0.02

Beta-blocker administration before and after operation and mean maximum sinus rate per day was not statistically different. The prevalence of postoperative atrial fibrillation was significantly less in the patients randomized to BAP group when compared with the other two remaining groups (Figure 1).

An episode of atrial fibrillation occurred in 7 (17.5%) of 40 patients in the BAP group compared with 12 (30%) of 40 patients in the LAP group (P=0.04), and 18 (45%) of 40 patients in control group (p=0.02). The first postoperative episode of atrial fibrillation occurred 2.5±1.3 days after surgery in LAP group, 2.4±1.6 days after surgery in control group, and 2.8±0.7 days afetr surgery in BAP group (p=0.5). The mean duration of atrial fibrillation were 6.9±4.2 hours in BAP, 6.1±2.8 hours in LAP and 7.2±1.6 in control group, (p=0.3).

If AF was not converted spontaneously to sinus rate (SR) in 48 hours either pharmacological means or electrical cardioversion was used to restore SR before discharge. The mean length of hospital stay was most significantly reduced in BAP group (6.1±1.2 versus 9.0±4.1 days in the control groups; p=0.01, and 8.7±1.3 days in LAP groups; p=0.02). The mean length of stay in the intensive care unit was also significantly reduced in the BAP group (2.8±0.7 versus 4.6±4.5 days in control group; p=0.04, and 4.2±3.2 days in LAP group; p=0.01). Cerebral events (including stroke or transient ischemic attacks) occurred in 4 patients and there was no significant difference between three groups. The mean hospital charges in the BAP was 6250$, in LAP was 6820$ and in control group was 7400$, (p=0.3). Postoperative complications in each group are presented in Table 2.

## Discussion

Previous studies demonstrated that use of beta-blockers and sotalol have beneficial effect in prevention of postopertive AF [5, 21]. Daoud and associates showed that preopertive amiodarone therapy was effective in reduction of postoperive AF [7]. Medical therapy as a prophylactic agent against post-CABG AF, may be limited by other medical disease, such as asthma, thyroid dsyfunction, or liver function derangement [20, 22]. Biatrial pacing has been shown to prevent of AF recurrence in patients with paroxysmal AF [18 –20, 23 –26]. Another study demonstrated that single-site pacing has not been effective in patients with AF [27]. Our study reported the comparision of biatrial  pacing with single-site atrial pacing and no pacing. Biatrial pacing prevents AF by two mechanism: a) The common cause of initiation of AF is premature atrial beat, especialy during sinus bradycardia. Biatrail pacing at a relatively high rate may result in supperession of atrial ectopy. b) Atrial conduction delay and dispresion of atrail refractoriess serve as a predictor for reentry and initiation of AF [7,19]. In our study, a triggered pacing mode was chosen to assure early activation of the atrial myocardium near the coronary sinus in response to premature atrial conduction sensed in either the right or left atria and hence reduce atrial dispresion.

Taylor et al showed that the most expensive complications post-CABG were respiratory failure and sternal wound infection, but occurred in only 3% and 0.4% of patients, respectively. However, AF was least expensive but most common complication, occurred in 20% of patients [28]. In our study hospital stay and hospital charges were significantly reduced by biatrial pacing, compared to single site or no pacing groups. Despite these benefits this technique was not associated with side effects. Identifying patients at risk for developing post-CABG AF and using biatrial pacing may be the optimal effective strategy.

## Conclusion

AF is commonly encountered post-CABG and, results in an increased length of hospital stay and cost. An ideal prophylactic approach is one that is effective in a diverse patient population and that is associated with minimal expense and risk[19]. Biatrail pacing may be such a technique, and is more effective in preventing of post-CABG AF than single-site atrial pacing and no pacing group and it results in a shortened length of hospitalization. This technique is not associated with a risk of ventricular arrhythmia, bradycardia, or hypotension, unlike antiarrhythmic agents. Identifying patients at risk for developing post-CABG AF and using biatrial pacing may be the optimal effective strategy.

## Figures and Tables

**Table 1:**
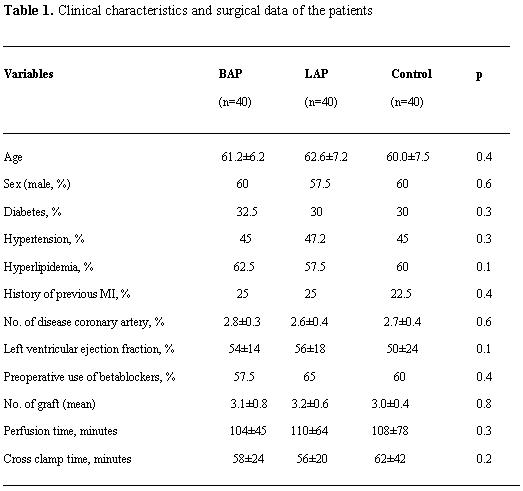
Clinical characteristics and surgical data of the patients

**Table 2:**
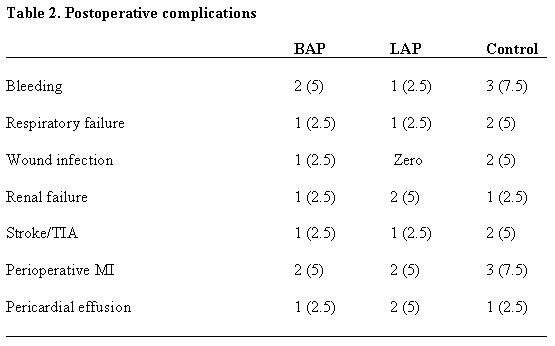
Postoperative complications
